# Improvement in Limit of Detection of Enzymatic Biogas Sensor Utilizing Chromatography Paper for Breath Analysis

**DOI:** 10.3390/s18020440

**Published:** 2018-02-02

**Authors:** Masanobu Motooka, Shigeyasu Uno

**Affiliations:** Department of Electrical and Electronic Engineering, Ritsumeikan University, Kusatsu, Shiga 525-8577, Japan; re0064vp@ed.ritsumei.ac.jp

**Keywords:** electrochemical sensor, biosensor utilizing paper, gas sensor, limit of detection

## Abstract

Breath analysis is considered to be an effective method for point-of-care diagnosis due to its noninvasiveness, quickness and simplicity. Gas sensors for breath analysis require detection of low-concentration substances. In this paper, we propose that reduction of the background current improves the limit of detection of enzymatic biogas sensors utilizing chromatography paper. After clarifying the cause of the background current, we reduced the background current by improving the fabrication process of the sensors utilizing paper. Finally, we evaluated the limit of detection of the sensor with the sample vapor of ethanol gas. The experiment showed about a 50% reduction of the limit of detection compared to previously-reported sensor. This result presents the possibility of the sensor being applied in diagnosis, such as for diabetes, by further lowering the limit of detection.

## 1. Introduction

In recent years, the number of deaths caused by chronic diseases has been increasing all over the world, and thus point-of-care (POC) diagnosis—one of the methods of preventative healthcare—has attracted attention [1,2,3]. As a POC diagnosis, breath analysis is an attractive method. It offers a noninvasive, rapid and simple diagnosis to patients, and can lead to early detection of chronic disease by monitoring health status on a daily basis. In addition, it has been proven that human breath contains various substances that have been established as biomarkers related to chronic diseases: acetone to diabetes, isoprene to blood cholesterol, etc. [4,5]. However, the biomarkers are included in human breath at low concentrations; sub parts per million (ppm) or parts per billion (ppb). For instance, the acetone concentration for diabetes is reported to be more than 1.8 ppm [6,7]. In addition, more than 1000 other volatile compounds are mixed with the biomarkers at the same concentration levels in human breath [5,8]. Therefore, the gas sensor for breath analysis requires a low limit of detection (LOD) and a high selectivity in order to detect the specific biomarkers.

The gold standard of analyzing biomarkers in human breath is gas chromatography mass spectrometry (GC-MS) [4,6,9,10]. While it offers high sensitivity and selectivity, it is mainly for laboratory use only, and therefore equipment size tends to be large. For POC applications, portable and small-sized gas sensors are desirable. An example of such small-sized gas sensors is the semiconductor-type sensor [11,12], which reads the change in electric resistance due to gas adsorption on the surface of the semiconductor film [13]. Although such sensors can have a high detection sensitivity, they have a problem in that they may provide unreliable results due to poor selectivity [14]. Various electrochemical gas sensors have also been reported [15,16,17,18]. However, many such electrochemical sensors have been developed mainly for toxic gases such as CO*_x_*, SO_2_, or NO_2_ and are not suitable for breath analysis applications. Optical or surface acoustic wave gas sensors have also been developed [17], but they have essentially the same problem of selectivity as semiconductor-type sensors.

To provide improved devices, studies of small-sized biogas sensors utilizing enzymes have been conducted for breath analysis [14,19]. As the enzymes have high substrate specificity, these sensors make it possible to detect only the target substances. A simple fabrication process to immobilize enzymes and mediators in chromatography paper has also been proposed [20]. The enzymes and mediators were held in sheets of chromatography paper and placed on a commercially available screen-printed carbon electrode. The sensor was able to read the electrochemical current derived from an enzymatic reaction in liquid phase under target gas exposure, and demonstrated successful detection of ethanol molecules in gas phase. Based on this report, an ethanol gas sensor fabricated only with chromatography paper has also been reported [21].

Such enzymatic biogas sensing methods utilizing paper could provide highly cost-effective disposable sensors for POC applications. Moreover, its principle can easily be applied to various target molecules by merely changing the enzymes and mediators. Improvements of the principle of ethanol gas sensors utilizing paper could eventually result in important insights in realizing/improving biogas sensors for other target gas molecules. From this perspective, we consider that the most important issue related to the present enzymatic biogas sensor utilizing paper proposed in [20,21] is its high LOD. Revealing a new and universal way to improve the LOD of enzymatic biogas sensor utilizing paper would contribute to realizing more challenging sensors, such as acetone sensors. 

In general, LOD can be improved in two ways. One is to increase sensitivity, and the other is to reduce standard deviation of the measured current at no target substance. While increasing sensitivity is straightforward, it would require increasing enzyme/mediator concentrations in each sensor, and therefore would sacrifice the cost-effectiveness of the sensor. On the other hand, reducing standard deviation of the current at zero target concentration, i.e., the background current (BGC), can be realized by improving the fabrication process while retaining the cost-effectiveness. 

In this study, we investigated the cause of background current in the enzymatic biogas sensor utilizing paper and attempted to reduce it by changing the fabrication and reagent preparation processes proposed by Kuretake et al. [20]. Then, we fabricated a new biogas sensor with reduced background current. Finally, we measured ethanol gas with the conventional and new biosensors to compare the limit of detection.

This paper consists of three sections; Section 2 describes the theory and experimental method, in Section 3 the results and discussion are shown, and Section 4 is the conclusion.

## 2. Theory and Experimental Method

### 2.1. Basic Principle of Limit of Detection

The LOD is defined as shown below [22]:

(1) $$C_{L}=\frac{{3S}_{B}}{m},$$

where *C*_L_ is the LOD (concentration), *S*_B_ is the standard deviation at no target substance (BGC), *m* is the detection sensitivity defined as the slope of the calibration curve. Assuming that the coefficient of variation (CV) is unchanged, *S*_B_ can be reduced by merely reducing the average value of the BGC [23,24]. In this work, we aim to reduce BGC to improve LOD. 

### 2.2. Ethanol Sensing Principle

**Figure 1:**
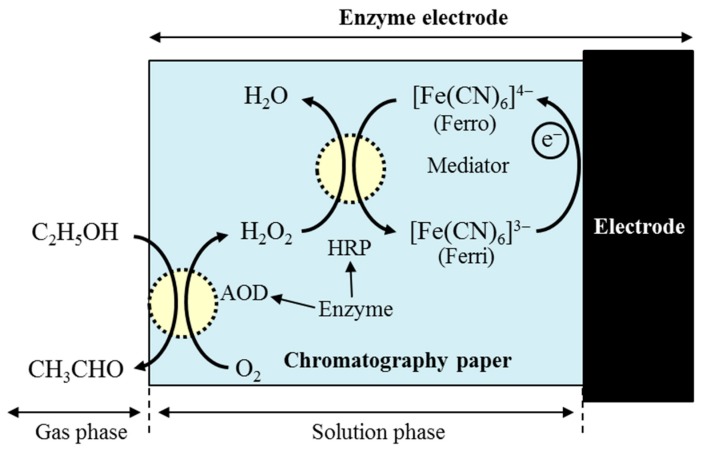
Schematic of the principle of reaction on the biogas sensor for ethanol gas measurement [20,21,25,26].

The principle of the biogas sensor using enzymes and mediator for an ethanol gas measurement is shown in Figure 1. By blowing ethanol gas on the biogas sensor, the following reactions occur. In the first reaction, ethanol (C_2_H_5_OH) is oxidized by dissolved oxygen (O_2_) due to substance specificity and catalysis of alcohol oxidase (AOD). Then the acetaldehyde (CH_3_CHO) and hydrogen peroxide (H_2_O_2_) are generated. The reaction is described as shown below:

C_2_H_5_OH + O_2_ → CH_3_CHO + H_2_O_2_. (2)

The H_2_O_2_ also oxidizes ferrocyanide ion ([Fe(CN)_6_]^4^^−^: Ferro) as the electron mediator by catalysis of peroxidase (HRP), where the ferricyanide ion ([Fe(CN)_6_]^3^^−^: Ferri) is generated. The reaction is described as:

H_2_O_2_ + 2[Fe(CN)_6_]^4^^−^ +2H^+^ → 2H_2_O + 2[Fe(CN)_6_]^3^^−^. (3)

The generated Ferri is reduced by receiving the electrons from the electrode under the applied negative potential (−0.2 V) with respect to the redox potential, finally generating the current. This is described as the following reaction:

2[Fe(CN)_6_]^3^^−^+ 2e^−^ → 2[Fe(CN)_6_]^4^^−^. (4)

The concentration of ethanol gas is quantified with Chronoamperometry measurement (CA), which reads the flowing current as a function of time under a potential step. The current value, *I*(*t*) (A), is described by the Cottrell’s equation as follows [27]:

(5) $$I(t)=\mathrm{nFAC}\sqrt{\frac{D}{\pi t}},$$

where *n* is the number of electrons, *F* (C/mol) is the Faraday constant, *A* (cm^2^) is the area of electrode, *C* (mol/cm^3^) is the concentration of substances, *D* (cm^2^/s) is the diffusion coefficient, and *t* (s) is the time after the potential application.

### 2.3. Fabrication of Ethanol Biogas Sensor

**Figure 2:**
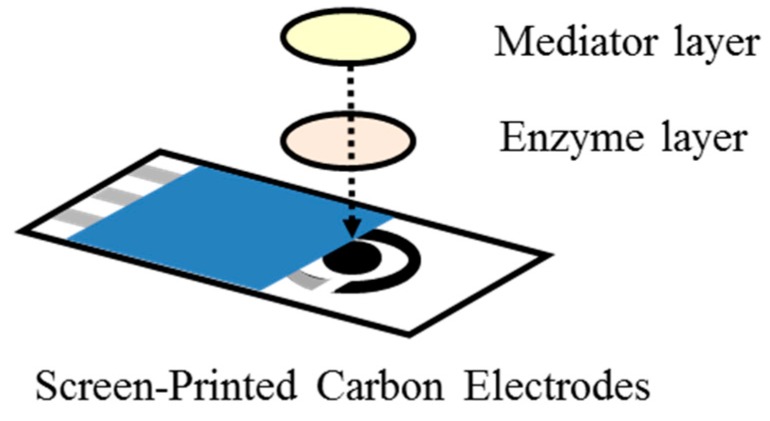
Structure of the biogas sensor (ChrSPCE) using chromatography papers with immobilized mediator and enzyme [20].

The structure of chromatography paper enzyme electrode (ChrSPCE) is schematically illustrated in Figure 2. The biogas sensor consists of Screen-printed Carbon Electrodes (SPCEs: DRP-110, DropSens, Asturias, Spain) and two layers; an enzyme layer and a mediator layer. The two layers are made of chromatography paper (Chrpr: No. 3001-878, whatman, GE Healthcare, Chicago, IL, USA), which were cut out in a circle with a diameter of 6.0 mm. For the enzyme layer, the Chrpr was immersed in the enzyme solution, and was dried for immobilization. The enzyme solution (AOD/HRP) was prepared by mixing phosphate buffer solution (PBS (mixing K_2_HPO_4_, KH_2_PO_4_): 100 mM, pH = 7.0, Wako, Osaka, Japan), alcohol oxidase (AOD: CAS RN 9073-63-6, from Pichia Pastris, Sigma-Aldrich, St.Louis, MO, USA), and peroxidase (HRP: No. 9003-99-0, from Horseradish, Wako). For the mediator layer, the Chrpr was also immersed in the solution including ferrocyanide (Ferro solution), which was prepared by mixing potassium ferrocyanide (K_4_[Fe(CN)_6_]: Wako) with PBS, and dried. The biogas sensor was fabricated by placing enzyme layer and mediator layer in this order on the SPCEs. These layers are hydrated by PBS prior to gas sensing to keep liquid phase with dissolved enzymes and mediators in the paper layers. The measured gas sample is blown onto the paper, and target molecules will diffuse into the liquid phase in the paper layers to generate enzymatic reaction. The mediator layer is placed above the enzyme layer to assist PBS absorption into the paper. This is possible because the mediator layer is more hydrophilic than the enzyme layer. 

### 2.4. Investigation of the Conditions for Reducing Background Current

In order to investigate the mechanism of BGC and the fabrication method for its reduction, several experiments are performed. In the Ferro solution used for the mediator layer in Figure 2, the Ferro and dissolved oxygen are mixed and the standard electrode potential are defined as follows [27]:

O_2_ + 4H^+^ + 4e^−^ = 2H_2_O    *E*° = 1.229V (vs. NHE), (6)

[Fe(CN)_6_]^3^^−^ + e^−^ = [Fe(CN)_6_]^4^^−^  *E*° = 0.361V (vs. NHE), (7)

where *E*° is the standard electrode potential, NHE is the normal hydrogen electrode. Owing to the potential difference, the following reaction proceeds in the process of drying and immobilizing the Ferro solution in the Chrpr:

4[Fe(CN)_6_]^4^^−^ + O_2_ + 4H^+^ → 4[Fe(CN)_6_]^3^^−^ + 2H_2_O. (8)

Therefore, the Ferri is generated even without the ethanol gas due to dissolved oxygen, and the BGC would occur. 

Based on this assumption, we measured the BGC depending on Ferro solution under a reduced amount of dissolved oxygen. The two kinds of Ferro solutions (A_1_, A_2_), where the amount of dissolved oxygen was varied by bubbling with nitrogen gas (PLL 41683161101763, GL Sciences, Tokyo, Japan) for 3 min, were prepared as shown in Table 1. Furthermore, we also investigated the condition of the Ferro solutions for further reduction of the BGC because it is difficult to remove the dissolved oxygen completely from the Ferro solution. The Ferro solutions under varying storage temperatures (B_1_, B_2_, B_2_), storage time (C_1_, C_2_, C_3_, C_4_), and Ferro concentrations (D_1_, D_2_, D_3_, D_4_) were prepared as in Table 1, and the BGC was measured.

The experimental system was set up by connecting a personal computer (PC), electrochemical analyzer (ALS: CH Instruments Electrochemical Analyzer, Model 6081E, BAS), and SPCEs. The Ferro solution 50 uL of each condition was dropped on the SPCEs, and then CA was performed for 50 s by applying −0.2V potential step vs. Ag/AgCl (reference electrode). The measurement was performed five times for each Ferro solution.

### 2.5. Sensor Testing with Ethanol Gas for Analysis of Limit of Detection 

Based on the insights obtained in the experiments described in Section 2.4, we aim to find the optimum fabrication conditions and perform ethanol gas sensing experiments. To do so, we fabricated two different sensors denoted as a-ChrSPCE and b-ChrSPCE for comparison. Both sensors have the same basic structure described in Section 2.3. The same enzyme layers fabricated with AOD/HRP 70 U/mL and dried at 4 °C for 12 h were used but different mediator layers were used, respectively. In a-ChrSPCE, the mediator layer was fabricated with Ferro 100 mM and dried at 4 °C for 12 h, which was based on conditions described by Kuretake [20]. In b-ChrSPCE, the mediator layer was fabricated with different condition described later in Section 3.1.2 to reduce the BGC. Ethanol gas sensing experiments were performed using these different sensors, and LOD was compared. The measurement system was set up by connecting PC, ALS, and each ChrSPCE. Before measurement of ethanol gas, the enzyme and mediator layers on the ChrSPCE were completely immersed in PBS 12 uL to prepare the solution phase for gas adsorption. The CA was performed by applying −0.2 V potential step vs. Ag/AgCl (Reference Electrode). The sample vapor of ethanol gas 20 mL was blown by a syringe pump on the wet biogas sensor in 20 s. As the paper layers are kept wet prior to gas exposure, the two pieces of papers are tightly adhered to the surface of SPCEs and are not dislocated, even under gas blow. Additionally, we confirmed that the hydration of the paper is not dried out by the gas blow. We measured the current by ethanol gas up to 200 s. The measurement with each ChrSPCE was performed five times for each concentration (0, 50, 100, and 150 ppm (*v*/*v*)), and replacing the enzyme and mediator layers per measurement.

## 3. Results and Discussion

### 3.1. Reduction of Background Current for Biogas Sensor

#### 3.1.1. Condition for Reducing Background Current

**Figure 3:**
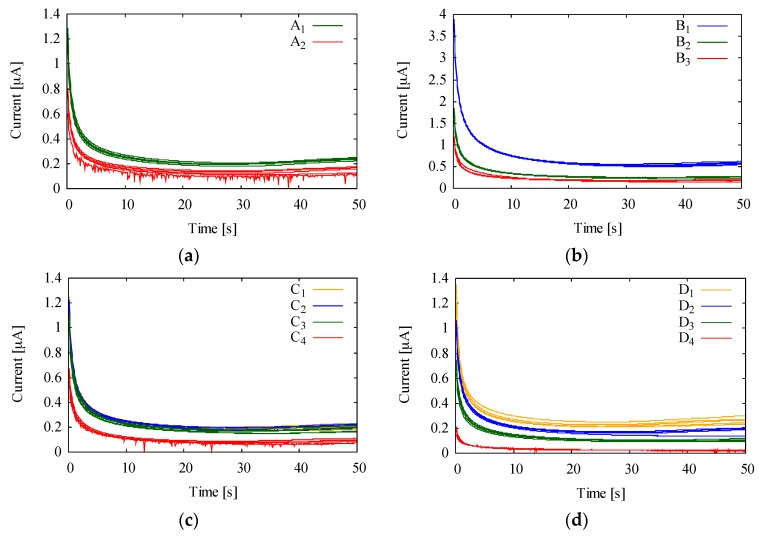
The background current (BGC) of the Ferro solution with varied conditions. (**a**) BGC with various amount of dissolved oxygen, (**b**) storage temperature, (**c**) storage time, (**d**) Ferro concentration.

Figure 3 shows the BGC of Ferro solution with CA under various conditions ((a) dissolved oxygen, (b) storage temperature, (c) storage time, (d) Ferro concentration). Figure 3a shows that the BGC of the Ferro solution is reduced by removing the dissolved oxygen. This indicates that dissolved oxygen is one of the causes for the BGC, which is consistent with the assumption in Section 2.4. Hence it is necessary to reduce the amount of dissolved oxygen for further reduction of the BGC in the sensor, and the BGC may be eliminated provided that the dissolved oxygen is completely removed. Figure 3b–d shows that the BGC of the Ferro solution was minimized, respectively, when stored at the lowest temperature, for the shortest time, and with the lowest concentration. The reduction of the BGC in Figure 3b–d can be explained by the following rate equation and Arrhenius equation [27]:

*v* = *k* [*X*], (9)

(10) $$k=A\;\exp\;[-\frac{E}{RT}],$$

where *v* (M/s) is the reaction rate, [*X*] (M) is the concentration of reactant, and *A* is the frequency factor, *E* (J/mol) is activation energy of a reaction, *R* (J/mol∙K) is the gas constant, *T* (K) is the absolute temperature. From Equations (9) and (10), the rate of reaction changed from Ferro to Ferri in (8) decreases at a lower temperature, in a shorter time, and with a lower concentration of the Ferro, leading to the reduction of the BGC due to the Ferri.

Ultimately, all the results in Figure 3 prove that it would be desirable to modify Chrpr with the Ferro solution for a shorter time, at lower temperature, at a lower concentration, and a lowered amount of dissolved oxygen. 

#### 3.1.2. Trade-Off between Different Parameters

When the Ferro solution is immobilized in Chrpr by drying, there is a trade-off between drying temperature and drying time, and it can be difficult to fabricate the mediator layer in a short time at low temperature. Therefore, we additionally measured using mediator layers under various drying conditions, and selected the mediator layer with the smallest BGC as new mediator layer. The conditions of the fabricated mediator layers are shown in Table 2. For the mediator layers, 3 mM of the Ferro solution with reduced dissolved oxygen by nitrogen gas as in Section 2.4 was dried in the Chrpr. The concentration of the Ferro solution is an optimum value calculated from an enzyme concentration of 70 U/mL. The mediator layer was placed on the SPCEs, and the 6 uL of PBS was dropped on it. Subsequently, the CA was performed under the same settings as in Section 2.4. 

**Figure 4:**
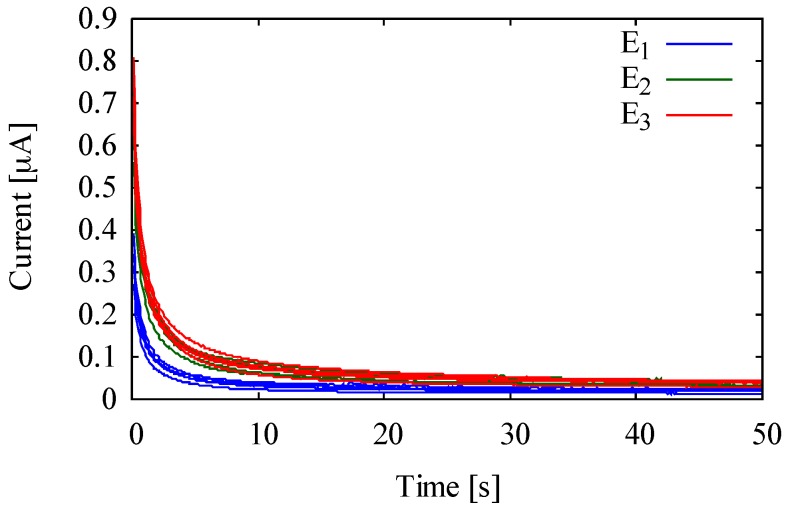
BGC of the mediator layer fabricated under various drying conditions.

Figure 4 shows the BGC of the mediator layers under E_1_, E_2_, and E_3_ shown in Table 2. The BGC of the mediator layer E_1_ dried at 40 °C in 20 min was the smallest. Based on this result, we used the mediator layer dried in the Chrpr at 40 °C in 20 min using 3 mM of the Ferro solution with a small amount of dissolved oxygen for the rest of this work.

### 3.2. Evaluation of Limit of Detection with Different Ethanol Biogas Sensors

**Figure 5:**
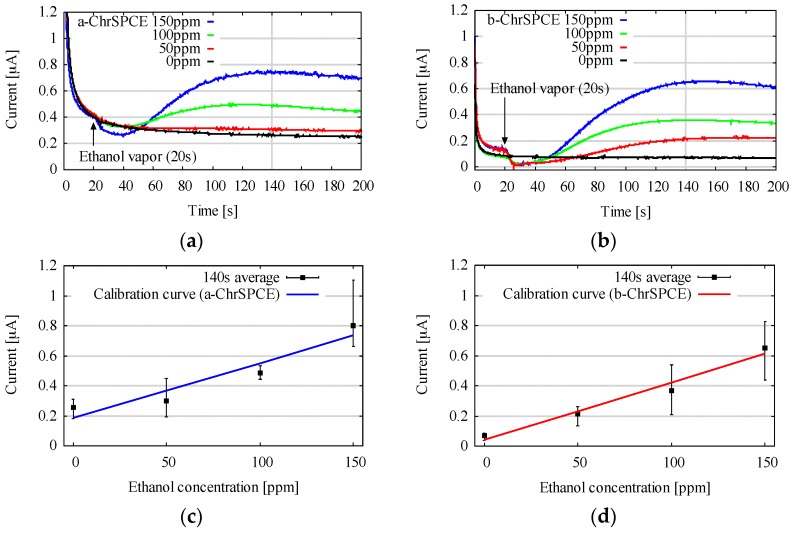
Representative current responses of a-ChrSPCE (**a**) and b-ChrSPCE (**b**) for several ethanol vapor concentrations: 0, 50, 100, 150 ppm (*v*/*v*). The calibration curves of the reduction current taken at *t* = 140 s in a-ChrSPCE (**c**) and b-ChrSPCE (**d**) of each ethanol gas (*Is*: output current [uA], *S*: ethanol concentration [ppm (*v*/*v*)]).

Based on the insights obtained from the results in Section 3.1.2, we fabricated our new biogas sensor, denoted as b-ChrSPCE, whereby mediator layer was fabricated at 40 °C in 20 min using 3 mM to reduce the BGC. The other sensor, denoted as a-ChrSPCE, was fabricated under the previously reported conditions [20] described in Section 2.5. They were tested as two kinds of ethanol gas sensor, and their performances were compared. Representative results of the CA with various gas concentrations (0, 50, 100, and 150 ppm (*v*/*v*)) using the two kinds of ChrSPCEs are shown in Figure 5a,b. Figure 5c,d indicates the calibration curves based on the currents at 140 s and the error bars from five independent measurements. From Figure 5a–d, the BGC can be observed in both a-ChrSPCE and b-ChrSPCE, as a result of the reduction reaction of Ferri by the working electrode. When the vapor of ethanol gas was blown onto each ChrSPCE, the current due to the enzymatic reaction was added to the BGC, and the linear response of the output current was observed between 0 and 150 ppm (*v*/*v*). The output current reached its maximum value at about 140 s for all concentrations. Table 3 summarizes the average value of BGC (*A*_BGC_), standard deviation (*S*_B_), slope of calibration curve (*m*), and resulting LOD (*C*_L_) of a-ChrSPCE and b-ChrSPCE. For b-ChrSPCE, the *A*_BGC_ was reduced from 0.25 to 0.071 uA compared to the a-ChrSPCE, and the *S*_B_ was also reduced from 0.048 uA to 0.019 uA. Considering that the mechanism of variability is the same for a-ChrSPCE and b-ChrSPCEs, the coefficient of variation (CV) might be the same, indicating that *S*_B_ will decrease as *A*_BGC_ decreases, as predicted theoretically in Section 2.1. On the other hand, the reduction in *A*_BGC_ did not affect *m*. This is attributed to the fact that the enzyme concentration, and therefore the resulting enzymatic current, was the same for a-ChrSPCE and b-ChrSPCE. Each *C*_L_ was calculated from Equation (1) based on each *S*_B_ and *m*, and decreased from about 40.000 ppm (39 ppm) in a-ChrSPCE to about 20.000 ppm (15 ppm) in b-ChrSPCE, which is about a 50% reduction in LOD. These results suggest that the LOD was, in fact, improved by reducing the BGC. 

In order to further improve the LOD, it is necessary to reduce the BGC by fabricating a more optimal mediator layer. The dissolved oxygen in the Ferro solution must be completely removed, and Chrpr may be dried with a special dryer (such as vacuum dryer or vacuum freezer dryer etc.) when fabricating the mediator layer. Then, the mediator layer would reduce the BGC considerably, and it would lower the LOD. Furthermore, optimizing the enzyme layer would improve the LOD. From Equation (1), the *C*_L_ decreases when increasing the *m*. There is a proportional relationship between enzyme concentration and enzyme reaction rate from Michaelis-Menten equation [28]; therefore, the current by enzyme reaction would increase at a higher concentration of the enzyme, leading to a high *m* and low *C*_L_. However, increasing enzyme concentration would lead to higher sensor cost, as pointed out in the introduction. In other words, there is a trade-off between cost-effectiveness and improved LOD by means of sensitivity.

Our findings indicate that the LODs of the other biogas sensors using Ferro as the mediator would also be improved in the same way [21]. Moreover, the LODs of the various biosensors using other types of the mediators may also be improved. For instance, ferrocene is frequently used as a mediator for biosensors [29,30]. The standard electrode potential is 0.771 V (vs. NHE), and this is smaller than the potential of dissolved oxygen (1.229 V (vs. NHE)). From the potential difference, therefore, the mediator should be degraded as Ferro due to the dissolved oxygen when modifying various electrodes with the mediator solution for sensor fabrication. Thus, the BGC of the sensors may reduce and the LODs are improved by optimizing the conditions of the immobilization for the mediator solution, as in this study. 

## 4. Conclusions

We attempted to reduce the background current to improve the LOD of a biogas sensor utilizing chromatography paper for ethanol. Ferro was oxidized by dissolved oxygen in the mediator layer of ChrSPCE due to the difference of the standard electrode potentials, which was responsible for the BGC of the sensor. We devised a new ChrSPCE for reduction of the BGC under various fabrication conditions (amount of dissolved oxygen, drying temperature, drying time, and concentration of the Ferro) of the mediator layer. As a result, the LOD of the sensor with the proposed ChrSPCE was reduced by around 50% compared to the sensor with conventional ChrSPCE. This study is a step toward detecting the low concentration substances with the biogas sensor, and it may be applied in diagnosing chronic diseases, such as diabetes, as a POC diagnosis sensor by further lowering the LOD. 

## Figures and Tables

**Table 1:** The Ferro solutions prepared with varied conditions (dissolved oxygen (A_1_, A_2_), storage temperatures (B_1_, B_2_, B_3_), storage times (C_1_, C_2_, C_3_, C_4_), and Ferro concentrations (D_1_, D_2_, D_3_, D_4_)) for reducing the BGC.

Ferro Solution Sample	N_2_ Bubbling	Storage Temperature (°C)	Storage Time (h)	Ferro Concentration (mM)
A_1_	—	4	12	100
A_2_	○	4	12	100
B_1_	—	24	12	100
B_2_	—	12	12	100
B_3_	—	4	12	100
C_1_	—	4	12	100
C_2_	—	4	9	100
C_3_	—	4	6	100
C_4_	—	4	3	100
D_1_	—	4	12	100
D_2_	—	4	12	60
D_3_	—	4	12	30
D_4_	—	4	12	3

**Table 2:** The mediator layers with various drying conditions for investigating the BGC.

Mediator Layer Sample	N_2_ Bubbling	Drying Temperature (°C)	Drying Time (min)	Ferro Concentration (mM)
E_1_	○	40	20	3
E_2_	○	24	120	3
E_3_	○	4	390	3

**Table 3:** BGC (*A*_BGC_), standard deviation (*S*_B_), slopes of calibration curve (*m*), and resulting LOD (*C*_L_) of two different sensors.

ChrSPCE	*A*_BGC_ (uA)	*S*_B_ (uA)	*M* (uA/ppm)	*C*_L_ (ppm)
a-ChrSPCE	0.25	0.048	3.7 × 10^-3^	40.000
b-ChrSPCE	0.071	0.019	3.8 × 10^-3^	20.000
